# A Meta-Analysis of the Impact of Intranasal Dexmedetomidine on Emergence Delirium and Agitation in Children and Adolescents Undergoing Tonsillectomy and/or Adenoidectomy

**DOI:** 10.3390/jcm14051586

**Published:** 2025-02-26

**Authors:** Abbas Al Mutair, Yasmine Alabbasi, Bushra Alshammari, Awatif M. Alrasheeday, Hanan F. Alharbi, Abdulsalam M. Aleid

**Affiliations:** 1 Department of Medical-Surgical Nursing, Princess Nourah bint Abdulrahman University, Riyadh 11671, Saudi Arabia; 2 Department of Maternity and Pediatric Nursing, College of Nursing, Princess Nourah bint Abdulrahman University, Riyadh 11671, Saudi Arabia; 3 Medical Surgical Nursing Department, College of Nursing, University of Hail, Hail 2440, Saudi Arabia; 4 Nursing Administration Department, College of Nursing, University of Hail, Hail 21424, Saudi Arabia; 5 Department of Surgery, Medical College, King Faisal University, Hofuf 31982, Saudi Arabia

**Keywords:** adenoidectomy, tonsillectomy, dexmedetomidine, children

## Abstract

**Background:** Tonsillectomy and adenoidectomy are two common pediatric operations that are frequently associated with postoperative problems like emergence agitation (EA) and emergence delirium (ED). Intranasal dexmedetomidine, which has anxiolytic and sedative qualities with low respiratory effects, is becoming increasingly popular as a premedication in pediatric patients. However, there is limited evidence on its efficacy in tonsillectomy and/or adenoidectomy. This original research is a meta-analysis examining the impact of intranasal dexmedetomidine on EA, ED, and other perioperative outcomes in children having these procedures. **Methods:** A thorough search of the PubMed, Scopus, Web of Science, and Cochrane Library databases was performed for randomized controlled trials (RCTs) published by January 2025 of select studies on children undergoing tonsillectomy and/or adenoidectomy. The intervention was intranasal dexmedetomidine (1–2 µg/kg), whereas the comparator was placebo/no intervention. **Results:** Four RCTs with 669 children met our inclusion criteria. Intranasal dexmedetomidine substantially decreased the incidence of EA (RR = 0.39, 95% CI: 0.16 to 0.92, *p* = 0.03) and ED (RR = 0.45, 95% CI: 0.24 to 0.84, *p* = 0.01), despite significant heterogeneity. Pediatric Anesthesia Emergency Delirium (PAED) scores were also considerably lower in the dexmedetomidine group (MD = −2.11, 95% CI interval: −3.77 to −0.44, *p* = 0.01). We found significant changes in extubation time (*p* = 0.91) or PACU discharge time (*p* = 0.53). **Conclusions:** Intranasal dexmedetomidine may reduce the occurrence of EA and ED, while also lowering PAED scores in children undergoing tonsillectomy and/or adenoidectomy. And although it has demonstrated safety with few side effects, more research is needed to validate its impact on other perioperative outcomes and enhanced dosing regimens.

## 1. Introduction

Adenoidectomy and tonsillectomy are the most common operations in children. Children may suffer anxiety or tension, and reluctance during procedures due to fear of pain, new operating room settings, and fasting, particularly during separation from parents. Transferring reluctant children to the surgery room may result in long-term psychological harm [1]. Children who are going to have surgery may exhibit acute worry or fear in the waiting area or during anesthesia induction. Preoperative anxiety in children has been linked to the greater use of analgesics, postoperative agitation, and behavioral abnormalities (e.g., feeding problems, sleep disorders, separation anxiety) [2,3].

Emergent agitation (EA) refers to poor postoperative behavior that may include symptoms such as aggressive movements, excitability, thrashing, disorientation, and sobbing [4]. The exact cause and pathophysiology of EA are unknown, although risk factors include preschool age, preoperative anxiety, postoperative pain, nausea, vomiting, otolaryngology operations, and inhalational anesthetics, particularly sevoflurane [5]. Emergence delirium (ED) is a behavioral illness in children marked by sobbing, panic, instability, and confusion during anesthetic recovery. Improper anesthetic procedures, as well as postoperative airway obstruction and discomfort, considerably increase the risk of complications [6,7]. General anesthesia, achieved via orotracheal intubation for airway management, carries inherent hazards. The majority of these complications are respiratory, resulting from laryngospasm and bronchial hyperresponsiveness, which may cause EA or ED that may manifest during or after the surgery [8]. Anesthesia research focuses on effective methods to promote cooperation, assure clinical safety, improve comfort, and prevent postoperative challenging outcomes. To effectively handle children’s perioperative anxiety, medical ethics and clinical practice must be considered simultaneously.

Studies have investigated medications used as premedication to minimize anxiety and smooth anesthesia induction [9,10]. The optimum premedication for children should be easily absorbed, have quick onset and offset, and cause minimal side effects. Previous studies showed that preoperative midazolam seemed to increase the incidence of perioperative respiratory adverse events [11]. Shen et al. reported that intranasal midazolam increased the risk of perioperative respiratory adverse events in children while dexmedetomidine decreased it [11]. Recent studies have explored alternative treatments and novel combinations to enhance pre-medication efficacy in pediatric patients undergoing tonsillectomy and/or adenoidectomy. A 2024 randomized controlled trial investigated the use of intranasal dexmedetomidine combined with esketamine compared to each drug alone. The study found that the combination significantly reduced the incidence of emergence delirium and postoperative negative behavioral changes, while also improving sedation quality and parental satisfaction, without notable adverse effects [12].

ERAS (Enhanced Recovery After Surgery) protocols advocate for multimodal analgesia, minimizing opioid use to reduce side effects that may contribute to ED and EA. The incorporation of agents like dexmedetomidine has been shown to be beneficial [13]. Intranasal dexmedetomidine is gaining popularity in pediatrics due to its anxiolytic qualities and minimal respiratory effects [11]. However, it may lead to delayed onset bradycardia, and hypotension [14].

According to previous studies, a single 84 μg dose of intranasal dexmedetomidine has a bioavailability of around 65% in healthy persons, with peak plasma levels arriving within 38 min. Intranasal administration takes longer to produce results than intravenous administration. A previous study found that giving dexmedetomidine intranasally at a dose of 1 µg/kg in children resulted in sedation within 25 min. These characteristics indicate that delivering intranasal dexmedetomidine 25 to 40 min before surgery may successfully produce sedation and minimize anxiety in children. The intranasal administration of sedatives has gained popularity due to the absence of the need for intravenous access and the effective delivery of the entire pre-calculated dose. The medication is administered directly to the nasal mucosa, thus circumventing hepatic first-pass metabolism, and plasma concentrations are potentially comparable to those of an intravenous dose [15].

It may also help to reduce postoperative agitation following minor procedures such as adenoidectomy and tonsillectomy without requiring an extended recovery period. However, the current literature lacks a comprehensive analysis of intranasal dexmedetomidine in children undergoing tonsillectomy and/or adenoidectomy. Thus, this study aims to determine the effects of intranasal dexmedetomidine on EA/ED in children undergoing tonsillectomy and/or adenoidectomy.

## 2. Methods

### 2.1. Study Registration

The study protocol was registered on PROSPERO (CRD42025640887). This systematic review and meta-analysis contained a PRISMA checklist in Supplementary File S1 and adhered to the Preferred Reporting Items for Systematic Reviews and Meta-Analyses (PRISMA) statement standards [16].

### 2.2. Literature Search and Study Selection

Without regard to language limitations, we carried out a thorough search of the Web of Science, Cochrane Library, PubMed, and Scopus databases for articles published up until 10 January 2025. The terms “Dexmedetomidine” and “intranasal” and “Children” and “Pediatrics”, as well as “tonsillectomy or adeniodectomy”, were used. Search strategies were displayed in the Supplementary File S1.

To determine which papers satisfied the inclusion and exclusion criteria, two different authors independently examined the search results. They also assessed each study’s qualifying criteria. After the initial screening of abstracts and titles, we acquired the complete texts of potentially pertinent papers for a more thorough analysis. Any disputes that arose throughout the procedure were settled by consensus between the authors and if any conflict persisted, the senior author resolved it.

### 2.3. Eligibility Criteria

Studies that met the following PICO criteria were included:

1. Population: children and adolescents (2–18) years old undergoing tonsillectomy and/or adenoidectomy surgery.
2. Intervention: intranasal DEX in a dose of 1 or 2 µg/kg.
3. Comparator: Placebo/no intervention.
4. At least one of the following outcomes: emergence agitation/emergence delirium, extubation time (min), time to discharge from PACU, Pediatric Anesthesia Emergency Delirium (PAED) Scale score, and adverse events.
5. Study design: randomized controlled trials (RCTs). We excluded observational studies to avoid bias related to them and provide more robust evidence from RCTs.

Studies that did not meet our PICO criteria were not conducted in target population. We excluded studies reporting incomplete data.

### 2.4. Data Extraction

Data from the selected research studies was extracted by two authors using an Excel spreadsheet. Sections on study design, place of origin, overview of included studies, and baseline parameters such as age, male sex, duration of surgery and anesthesia, intervention, and control doses are all included in the data extraction form. Merging agitation/delirium, extubation time (minutes), time to discharge from the PACU, score on the PAED Scale, and adverse events were among the study’s outcomes. Consensus was used to resolve disagreements.

### 2.5. Quality Assessment

Two authors used the Cochrane tool for RCTs (ROB2) [17] to evaluate the quality of the included studies. The Robvis web tool, RoB 2 for cluster-randomized trials (22 August 2019 version) was used to create quality assessment figures [18]. Domains assessed included randomization, deviation from intended intervention, missing outcome data, measurement of the outcome, and selection of the reported results. Discussion was used to resolve any disputes.

### 2.6. Outcome Data Measurement

One study rated EA on a four-point scale, while another used a five-point approach. Our research identified EA episodes with a score of three or above.

### 2.7. Statistical Analysis, Sensitivity Analysis, and Trial Sequence Analysis

The software we utilized for our statistical study was Review Manager 5.4. [19]. Risk ratios (RR) were used to evaluate binary outcomes, whereas mean difference (MD), with a 95% confidence level, was used to analyze continuous variables. We employed a random effects model in the outcome analysis to take variability into consideration. Significant heterogeneity was indicated by I2 values greater than 50%. *p*-values below 0.05 were regarded as statistically significant. Sensitivity analysis using the leave-one-out method was conducted to investigate the study that caused the heterogeneity using Review Manager. Trial sequential analysis (TSA) was calculated by R software 4.2.2. The analysis calculates cumulative effect sizes, such as odds ratios (ORs) or MD, as new studies are included. TSA also determines the Required Information Size (RIS), which indicates the sample size necessary to detect a true treatment effect with a predefined level of significance and power. Additionally, it tracks cumulative sample size, which reflects the total number of participants across studies, and cumulative Z-scores, which help assess the statistical significance of the cumulative results. Quality of evidence was assessed using Grading of Recommendations, Assessment, Development, and Evaluations (GRADE).

## 3. Results

### 3.1. Literature Search

**Figure 1:**
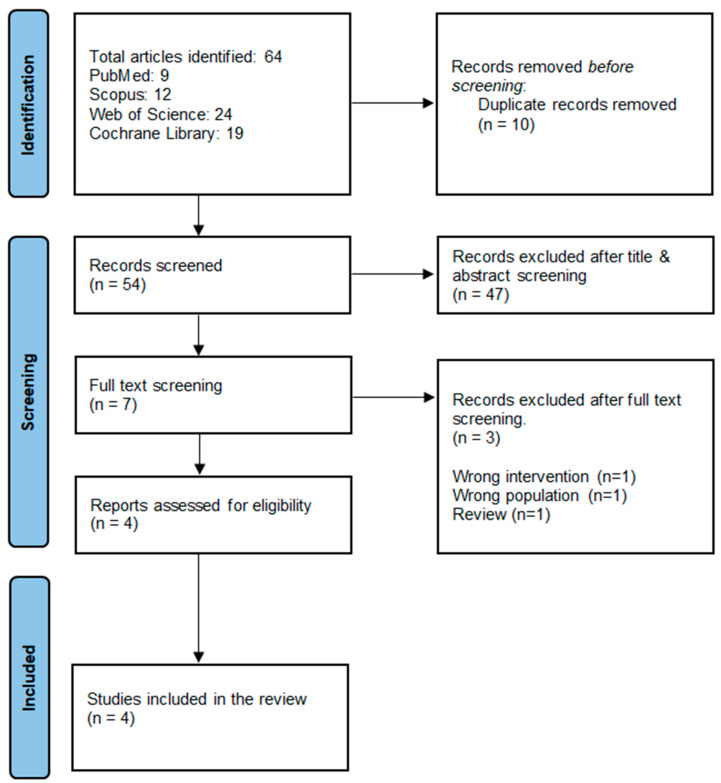
PRISMA flow chart of searching and screening processes.

A thorough literature search through PubMed, Scopus, Web of Science, and Cochrane Library was conducted and found 64 articles, 10 of which were duplicates. The remaining 54 records were screened for title and abstract, and 47 were excluded because they did not fit the inclusion criteria. Three of the seven publications that underwent full-text screening were excluded, leaving four studies acceptable for inclusion. (Figure 1).

### 3.2. Study Characteristics

We included four randomized clinical trials in our review [11,20,21,22]. A total of 669 children younger than 12 years were included in the systematic review with a sex ratio of 387:282 (male:female). The mean surgery duration was 34.65 ± 5.66, and the mean duration of anesthesia was 42.48 ± 5.68. The mean ± SD weight of patients ranged from 17.4 ± 3.4 kg to 20.9 ± 4.5 kg. Three studies were conducted in China [11,21,22], and only one study was carried out in Egypt [20]. The characteristics of the included studies are presented in Table 1.

### 3.3. Quality Assessment

**Figure 2:**
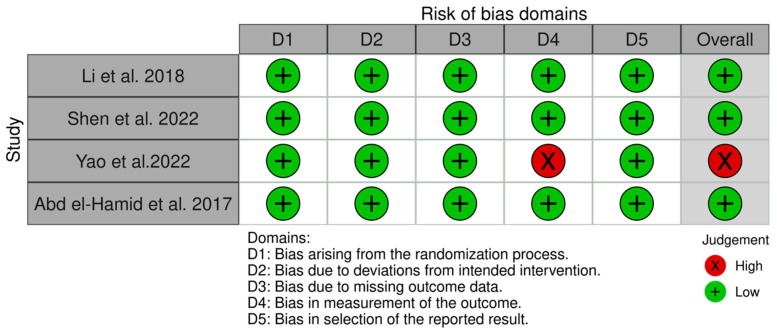
Risk of bias assessment of the included studies using Rob-2 tool [11,20,21,22].

The included studies yielded low quality in all domains, which indicate outstanding methodology [11,20,21]. Only Yao et al. had a high risk of bias [22]. Figure 2 shows the traffic-light plot of the quality assessment.

#### 3.3.1. Primary Efficacy Outcomes: Emergence Agitation and Delirium

**Figure 3:**
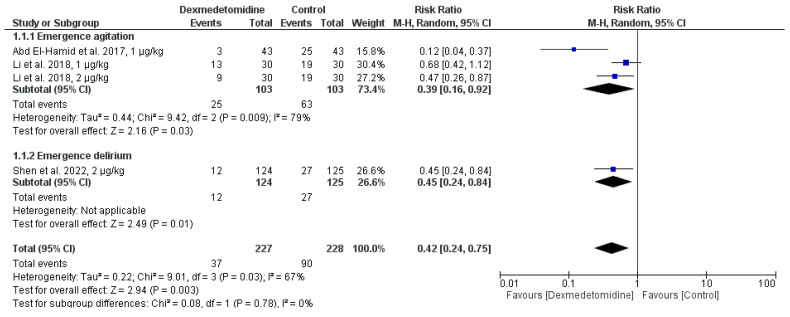
Incidence of Emergence Agitation and Delirium among patients taking dexmedetomidine and control groups [11,20,21].

Two studies compared the dexmedetomidine group’s incidence of emergence agitation to that of the control group. Although there was high heterogeneity (I2 = 79%, *p* = 0.009), the results showed that dexmedetomidine significantly decreased the incidence of emerging agitation (RR = 0.39, 95% CI: 0.16 to 0.92, *p* = 0.03) (Figure 3).

Dexmedetomidine significantly decreased the probability of emergence delirium, according to one study that evaluated its effects on the condition (RR = 0.45, 95% CI: 0.24 to 0.84, *p* = 0.01). With substantial heterogeneity (I2 = 67%, *p* = 0.03), the overall impact showed that dexmedetomidine significantly decreased the combined risk of these incidents (RR = 0.42, 95% CI: 0.24 to 0.75, *p* = 0.003) (Figure 3).

#### 3.3.2. Secondary Efficacy Outcomes

**Figure 4:**
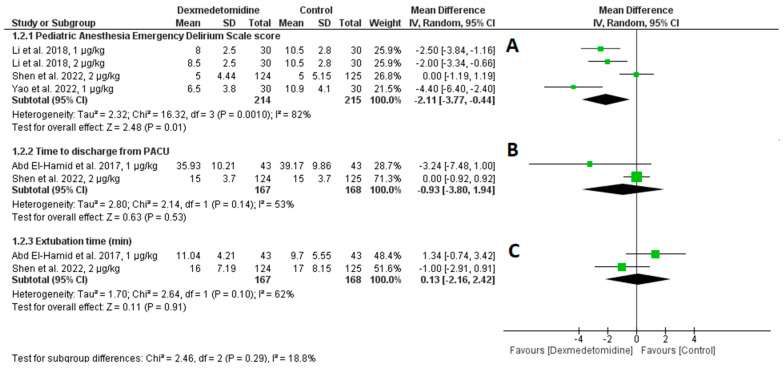
(**A**) Pediatric Anesthesia Emergency Delirium (PAED) Scale (**B**) Time to Discharge from PACU (**C**) Extubation Time in Minutes among patients taking dexmedetomidine and control groups [11,20,21,22].

Three studies evaluated the scores after surgery using the Pediatric Anesthesia Emergency Delirium (PAED) Scale. With substantial heterogeneity (I2 = 88%, *p* = 0.001), dexmedetomidine significantly reduced the PAED scale scores as compared to a control (Mean Difference [MD] = −2.11, 95% CI: −3.77 to −0.44, *p* = 0.01) (Figure 4A).

Two studies assessed the time to discharge from the post-anesthesia care unit (PACU). There was substantial heterogeneity (I2 = 53%, *p* = 0.14) and no discernible difference between dexmedetomidine and the control (MD = −0.93, 95% CI: −3.80 to 1.94, *p* = 0.53) (Figure 4B).

Two trials were included in the analysis for extubation time. The results showed considerable heterogeneity (I2 = 62%, *p* = 0.10) and no significant difference between dexmedetomidine and the control (MD = 0.13, 95% CI: −2.16 to 2.42, *p* = 0.91) (Figure 4C).

#### 3.3.3. Safety Outcomes: Adverse Events

According to Abd El-Hamid et al. [20], there was no statistically significant difference in nausea or vomiting between the two groups. The incidence of vomiting was similar in the other three trials that were included (*p* > 0.05).

#### 3.3.4. Sensitivity Analysis

By conducting sensitivity analysis using the leave-one-out method to remove one study at a time, we observed that Abd El-Hamid et al. [20] was the main source of heterogeneity in EA outcome due to the performance of this study in Egypt (different baseline characteristics such as lower weight and duration of surgery) and the other two included studies belong to the same population in the same study. In the pediatric anesthesia emergency delirium scale score, Yao et al. [22] was the main source of heterogeneity, and this may be attributed to different surgery (no adenoidectomy) and different dose.

#### 3.3.5. TSA of Emergence Agitation

The TSA for emergence agitation examines the effect of different doses of experimental treatments on the incidence of agitation following anesthesia. The analysis includes three studies: Abd El-Hamid et al. 2017 [20], 1 µg/kg, Li et al. 2018 [21], 1 µg/kg, and Li et al. 2018, 2 µg/kg [21]. The cumulative ORs from these studies suggest that the experimental treatments reduce the odds of emergence agitation compared to the control group, with all OR values being less than 1. This indicates a beneficial effect of the treatments in preventing agitation. Specifically, the OR for Abd El-Hamid et al. [20] is 0.054, and for the two doses in Li et al., the ORs are 0.155 and 0.181, respectively. These values show a trend toward a stronger effect with more data from subsequent studies.

However, despite this trend, the cumulative Z-scores for all three studies remain negative, with values of −4.33, −4.36, and −5.04, respectively. These negative values indicate that the cumulative odds ratios are not statistically significant, as they fall below the threshold for significance (Z < −1.96). This suggests that the evidence, while trending in favor of the experimental treatments, is not yet strong enough to confirm a statistically significant effect at the 95% confidence level.

The RIS indicates the sample size necessary to detect a reliable effect with sufficient power. For the first study (Abd El-Hamid et al. 2017 [20], the RIS is very low at 0.418, suggesting that the study already provides sufficient data. However, the Li et al. 2018 studies have higher RIS values (3.302 and 1.221) [21], indicating that larger sample sizes are needed in those cases to achieve statistical significance.

Finally, the cumulative sample sizes show that the number of participants increases with each study, from 86 in the first study to 206 in the third, gradually improving the power and reliability of the analysis. Overall, while the TSA suggests a potential benefit of the experimental treatments, further studies with larger sample sizes are required to definitively confirm the effect and achieve statistical significance in preventing emergence agitation.

#### 3.3.6. TSA of Intervention’s Effect on Delirium

The TSA results for studies assessing the intervention’s effect on delirium suggest that, cumulatively, the intervention does not demonstrate a statistically significant reduction in delirium compared to the control group. The cumulative effect sizes across the studies remain negative, with the final effect size stabilizing at approximately −0.70. This indicates that, on average, the intervention group had lower mean values than the control, but the difference does not appear to be clinically or statistically meaningful.

The RIS, which represents the number of participants needed to reach reliable conclusions, varies significantly between studies. For Li et al. 2018 (1 µg/kg and 2 µg/kg) and Yao 2022 (1 µg/kg), the RIS values are within a feasible range, suggesting that these studies contribute meaningfully to the analysis [21]. However, Shen 2022 (2 µg/kg) has an infinite RIS, likely due to high variability in the data or an effect size close to zero, which makes it difficult to determine a definitive conclusion from that study alone.

The cumulative sample size increases progressively across the included studies, reaching a total of 429 participants. Shen et al.’s 2022 study, with its large sample size, contributes significantly to this total. Despite this increase, the cumulative Z-scores remain below the critical threshold of ±1.96 required for statistical significance, with the final Z-score being −1.83 [11]. This suggests that, even when considering all available data, there is insufficient evidence to confirm that the intervention effectively reduces delirium.

### 3.4. Quality of Evidence

Using GRADE assessment, emergence agitation and delirium had a moderate certainty of evidence. Meanwhile, PAED scale scores, time to discharge, and extubation time had low certainties. (Table 2)

## 4. Discussion

Our meta-analysis assessed the safety and efficacy of Dexmedetomidine in children undergoing tonsillectomy and/or adenoidectomy. Dexmedetomidine demonstrated significant efficacy in reducing EA/ED, with significant reduction in PAED scale scores, suggesting its ability to mitigate agitation and improve patient recovery outcomes. However, there were comparable results regarding discharge time from the PACU or extubation time. Additionally, dexmedetomidine exhibited a favorable safety profile, with no significant differences in adverse events such as nausea and vomiting compared to control.

In clinical practice, children’s preoperative sedation and analgesia pose substantial challenges. Utilizing the high vascularity of the nasal mucosa, intranasal premedication has gained popularity as a noninvasive and successful method that allows for quick drug absorption while avoiding first-pass metabolism with few side effects.

When given 45–60 min prior to induction, Akin et al. found no discernible difference in the incidence and severity of EA when comparing the same dose and route of dexmedetomidine with intranasal midazolam [23]. Abd El-Hamid et al. [20] administered preoperative intranasal dexmedetomidine at a dose of 1 µg/kg in order to assess EA in children having tonsillectomy and/or adenoidectomy after sevoflurane anesthesia [20]. Authors reported that the incidence of EA was significantly lower in children allocated to the dexmedetomidine group (6.98%) compared to the placebo (58.14%), which reached statistical significance (*p* = 0.001). It should be mentioned that the primary outcome of the Abd El-Hamid et al. investigation was the detection of EA incidence. Dexmedetomidine’s sedative effects may be due to stimulation of α2-adrenoreceptors in the locus coeruleus, resulting in sleep-like EEG patterns [20].

In a recent trial, Liao et al. investigated the combination of intranasal dexmedetomidine and esketamine for premedication, comparing its effectiveness in decreasing complications to either drug alone in children undergoing tonsillectomy and/or adenoidectomy with sevoflurane anesthesia [12]. The findings confirmed dexmedetomidine’s efficacy in reducing ED, a finding consistent with the study conducted by Shen et al., who reported a significant reduction in ED after administration of dexmedetomidine compared to placebo (*p* < 0.017) [11].

A previous meta-analysis indicated that the use of dexmedetomidine in pediatric anesthesia was beneficial in reducing EA, decreasing PAED scores, shortening PACU stay lengths, reducing the need for additional anesthetics, and increasing parental satisfaction when compared to the placebo group [24]. Authors reported that a DEX dose of 2 µg/kg has the most significant effect in reducing the occurrence of EA. Lower doses of DEX (0.5 µg/kg) were linked to increased agitation rates. Thus, higher dosages of dexmedetomidine may be desirable in clinical settings to reduce EA under controlled conditions. Furthermore, as reported in our study, their data indicate that 1 µg/kg DEX significantly lowered PAED scores [24], which may be related to its neuroprotective characteristics, as demonstrated by recent studies [25]. Dexmedetomidine, an α2-adrenoceptor agonist [26], promotes neuroprotection by affecting neuroinflammation, apoptosis, oxidative stress, and synaptic plasticity via both α2-adrenoceptor-dependent and independent pathways.

Intranasal dexmedetomidine substantially decreased the occurrence of perioperative respiratory adverse events, specifically oxygen desaturation and coughing [11]. This positive effect could be attributed to a variety of processes. Importantly, dexmedetomidine most likely increased the degree of anesthesia, which reduced airway reflexes [27,28]. Further, its direct action on airway smooth muscle may have had an impact. Furthermore, individuals receiving dexmedetomidine required less fentanyl, most likely because of its modest analgesic effects [29]. Because intravenous fentanyl is known to produce coughing and respiratory depression [30], the lesser usage of fentanyl in the dexmedetomidine group might have been related to the reduced rate of coughing and desaturation.

There is increasing interest in Remimazolam, a new ultra-short-acting benzodiazepine with a favorable pharmacokinetic profile, for reducing the incidence of postoperative delirium. Unlike midazolam, Remimazolam undergoes rapid metabolism by tissue esterases, leading to a predictable onset, faster recovery, and reduced risk of accumulation, even in prolonged administration. Recent evidence [31,32] highlights Remimazolam’s potential in both adult and pediatric populations. Studies suggest that its minimal impact on hemodynamics and lower incidence of respiratory depression compared to other sedatives may contribute to a reduced risk of ED and postoperative cognitive dysfunction.

Given its safety and efficacy, integrating Remimazolam into the anesthetic regimen for pediatric tonsillectomy/adenoidectomy could be an area of future research, particularly in comparison to intranasal dexmedetomidine.

The use of ketamine as an adjunct for treating ED remains a topic of clinical and academic interest. Although ketamine is conventionally used as an anesthetic, emerging evidence supports its role in sedation strategies, particularly in cases of refractory EA. Pathophysiologically, ketamine acts as an NMDA receptor antagonist, modulating glutamate transmission and reducing hyperexcitability in the central nervous system. This mechanism aligns with the hypothesis that excessive cortical arousal contributes to emergence agitation. Sub-anesthetic doses (bolus + continuous infusion) have been successfully utilized in pediatric patients to stabilize emergence, particularly in the absence of organic causes such as low cardiac output syndrome (LCOS) or hyperlactacidemia. Current evidence supports a reduced incidence of severe ED when ketamine is used in small doses postoperatively and synergistic effects when it is combined with dexmedetomidine, enhancing sedation without excessive respiratory depression. It also has potential neuroprotective properties, particularly in younger patients at risk of long-term neurocognitive disturbances [33,34].

Comparable adverse events reported among the included trials [33,34], such as nausea and vomiting, further support its usage as a preoperative medication to decrease EA/ED in the pediatric population. Future well-designed large-scale RCTs are needed to draw more robust evidence and support our findings.

### Strengths and Limitations

This is, to our knowledge, the first comprehensive systematic review and meta-analysis to assess the safety and efficacy of nasal dexmedetomidine in children undergoing tonsillectomy and/or adenoidectomy. However, the small sample size and limited number of included studies may limit the generalizability of our findings. Heterogeneity in some outcomes acts as a major limitation, but it can be explained by the variety of doses, baseline characteristics, and surgeries. Additionally, most of the included studies were conducted in China, which may introduce regional biases related to differences in healthcare practices, patient demographics, and perioperative care protocols. These factors should be considered when applying findings to broader, more diverse populations. Furthermore, there is insufficient exploration of how these findings could be translated into daily clinical practice or what modifications might be necessary for different healthcare settings due to the aforementioned limitations. The significant variation identified among studies emphasizes the need for additional standardized research to validate these findings and determine the most effective dose and application practices. Future studies should prioritize multicenter, internationally representative trials to enhance the external validity of results. Additionally, refining methodologies, including standardized sedation scales and uniform dosing protocols, will help ensure consistency and improve clinical applicability. The integration of novel pharmacological agents such as remimazolam and adjunct ketamine therapy into ERAS protocols could significantly refine pediatric anesthesia practices. Further RCTs are needed to determine optimal dosing, routes of administration, and combination strategies to balance safety with efficacy.

## 5. Conclusions

Our findings showed that dexmedetomidine is an effective drug for managing emergence agitation and delirium, with a proven safety profile in children undergoing tonsillectomy and/or adenoidectomy. More well-designed and adequately powered RCTs are needed to support these findings.

## Figures and Tables

**Table 1:** Studies and Baseline Characteristics * Data are reported as range while other data reported as mean ± SD.

Study	Country	Study Type	Intervention	Sample Size	Age(y) *	Males, *n* (%)	Weight (kg), Mean ± SD	Type of Surgery			Duration of Surgery(min)	Duration of Anesthesia (min)
								Adenoidectomy	Adenoidectomy and Tonsillectomy	Tonsillectomy		
Li et al. 2018 [21]	China	Double-blind randomized controlled trial	dexmedetomidine (1.0 μg/kg)	30	4.47 ± 1.17	16 (53.3)	19.82 ± 5.51	13	17	-	36.87 ± 20.06	45.87 ± 20.29
			Dexmedetomidine (2.0 μg/kg)	30	4.53 ± 1.55	14 (46.7%)	20.05 ± 5.79	9	21	-	40.03 ± 17.33	48.60 ± 17.35
			0.9% saline	30	4.37 ± 1.30	20 (66.7%)	18.67 ± 4.10	12	18	-	34.10 ± 15.65	43.77 ± 16.11
Shen et al. 2022 [11]	China	Double-blind randomized controlled trial	Midazolam (0.1 mg/kg)	124	0–12 *	75 (60.5)	-	21	101	2	40 ± 15	47.9 ± 21.6
			Dexmedetomidine (2.0 μg/kg)	124	0–12 *	74 (60)	-	28	95	1	35 ± 15	40 ± 15
			0.9% saline	125	0–12 *	72 (58.1)	-	25	96	4	38.3 ± 18.75	45 ± 18.75
Yao et al.2022 [22]	China	Double-blind randomized controlled trial	dexmedetomidine (1.0 μg/kg)	30	4.4 ± 1.2	18 (60)	18.4 ± 4.9	-	14	16	36.00 ± 7.29	NA
			parental presence intervention and intranasal dexmedetomidine (1.0 μg/kg)	30	4.6 ± 1.4	16 (53.3)	19.7 ± 5.3	-	19	11	37.77 ± 8.89	NA
			parental presence intervention only	30	4.6 ± 1.2	18 (60)	20.9 ± 4.5	-	15	15	36.40 ± 7.20	NA
			Control	30	4.3 ± 1.1	20 (66.7)	19.9 ± 4.5	-	19	11	34.87 ± 1.03	NA
Abd el-Hamid et al. 2017 [20]	Egypt	Double-blind randomized controlled trial	dexmedetomidine (1.0 μg/kg)	43	4.4 ± 1.3	25 (58.1)	17.4 ± 3.4	2	31	10	22.4 ± 5.2	33.6 ± 6.5
			0.9% saline	43	4.2 ± 0.93	19 (44.2)	18.6 ± 4.1	3	28	12	24.1 ± 4.8	35.1 ± 5.9

SD: standard deviation, NA: not applicable.

**Table 2:** Grading of recommendations assessment, development, and evaluation (GRADE) evidence profile.

Certainty Assessment	Study Event Rates (%)	Effect	Certainty *	*n* of Studies	Study Design	RoB	Inconsistency	Indirectness	Imprecision	Others
Emergence Agitation	Dexmedetomidine vs. Control	RR 0.39 (0.16 to 0.92)	⨁⨁⨁◯ Moderate	2	RCTs	not serious	serious	not serious	not serious	none
Emergence Delirium	Dexmedetomidine vs. Control	RR 0.45 (0.24 to 0.84)	⨁⨁⨁◯ Moderate	1	RCTs	not serious	serious	not serious	not serious	none
Emergence Agitation and Delirium	Dexmedetomidine vs. Control	RR 0.42 (0.24 to 0.75)	⨁⨁⨁◯ Moderate	3	RCTs	not serious	serious	not serious	not serious	none
PAED Scale Scores	Dexmedetomidine vs. Control	MD −2.11 (−3.77 to −0.44)	⨁⨁⨁◯ Moderate	3	RCTs	not serious	very serious	not serious	serious	none
Time to Discharge from PACU	Dexmedetomidine vs. Control	MD −0.93 (−3.80 to 1.94)	⨁⨁◯◯ Low	2	RCTs	not serious	serious	not serious	serious	none
Extubation Time	Dexmedetomidine vs. Control	MD 0.13 (−2.16 to 2.42)	⨁⨁◯◯ Low	2	RCTs	not serious	serious	not serious	very serious	none

* Certainty: ⨁⨁◯◯ (low), ⨁⨁⨁◯ (moderate).

## Data Availability

The original contributions presented in the study are included in the article, further inquiries can be directed to the corresponding author.

## Supplementary Materials

The following supporting information can be downloaded at: www.mdpi.com/article/10.3390/jcm14051586/s1, File S1: PRISMA Checklist; File S2: Systematic Review Protocol.
