# Supplementary material for: A Meta-Analysis of the Impact of Intranasal Dexmedetomidine on Emergence Delirium and Agitation in Children and Adolescents Undergoing Tonsillectomy and/or Adenoidectomy
Source: J Clin Med. 2025 Feb 26;14(5):1586. doi: 10.3390/jcm14051586 (PMC11900937; doi:10.3390/jcm14051586)
Supplement: Supplementary file 1 [file jcm-14-01586-s001.zip › File S2.pdf]

Systematic Review and Meta-Analysis Protocol

TITLE

A Meta-Analysis of the Impact of Intranasal Dexmedetomidine on Emergence Delirium and Agitation in Children and Adolescents Undergoing Tonsillectomy and/or Adenoidectomy

AUTHORS

Abbas Al Mutair [1], Yasmine Alabbasi [2,*], Bushra Alshammari [3], Awatif M. Alrasheeday [4], Hanan F. Alharbi [2] and Abdulsalam M. Aleid [5]

[1] Department of Medical-Surgical Nursing, Princess Nourah bint Abdulrahman University, Riyadh 11671, Saudi Arabia
[2] Department of Maternity and Pediatric Nursing, College of Nursing, Princess Nourah bint Abdulrahman University, Riyadh 11671, Saudi Arabia
[3] Medical Surgical Nursing Department, College of Nursing, University of Hail, Hail 2440, Saudi Arabia
[4] Nursing Administration Department, College of Nursing, University of Hail, Hail 21424, Saudi Arabia
[5] Department of Surgery, Medical College, King Faisal University, Hofuf 31982, Saudi Arabia
* Correspondence: yaalabbasi@pnu.edu.sa

OBJECTIVES/ AIM OF STUDY

Objective: To systematically review and analyze existing literature on the efficacy of intranasal dexmedetomidine in managing emergence agitation (EA) and emergence delirium (ED) in pediatric patients undergoing tonsillectomy and/or adenoidectomy.

Aim: To provide a comprehensive evaluation of the impact of intranasal dexmedetomidine on perioperative outcomes, including EA, ED, Pediatric Anesthesia Emergency Delirium (PAED) scores, extubation time, PACU discharge time, and adverse events.

PICO

P (Population): Children undergoing tonsillectomy and/or adenoidectomy.

I (Intervention): Intranasal dexmedetomidine (1–2 µg/kg).

C (Comparison): Placebo/no intervention.

O (Outcome): Primary outcomes include the incidence of EA and ED. Secondary outcomes include PAED scores, extubation time, PACU discharge time, and adverse events.

KEYWORDS

Tonsillectomy, adenoidectomy, dexmedetomidine, emergence agitation, emergence delirium, pediatric anesthesia.

SEARCH STRATEGY

("Dexmedetomidine" AND "intranasal") AND ("Children" OR "Pediatrics") AND ("Tonsillectomy" OR "Adenoidectomy") AND ("Randomized Controlled Trial" OR "RCT" OR "Clinical Trial")

INCLUSION CRITERIA

Studies involving children undergoing tonsillectomy and/or adenoidectomy.

Studies examining intranasal dexmedetomidine interventions with at least one reported outcome of EA, ED, PAED scores, extubation time, PACU discharge time, or adverse events.

Randomized controlled trials (RCTs).

Studies published without language restrictions.

EXCLUSION CRITERIA

Studies not reporting primary or secondary outcomes of interest.

Non-randomized studies (e.g., observational studies, editorials, reviews).

Studies with incomplete data.

Studies not isolating results of intranasal dexmedetomidine as a single intervention.

DATA EXTRACTION VARIABLES

Study Characteristics: Author, country, study design, sample size, duration of surgery and anesthesia.

Patient Characteristics: Age, sex, baseline clinical features.

Intervention Characteristics: Dosage of intranasal dexmedetomidine.

Primary Outcomes: Incidence of EA and ED.

Secondary Outcomes: PAED scores, extubation time, PACU discharge time, adverse events.

Statistical Analysis: Effect size (RR, MD), confidence intervals, heterogeneity (I^2), publication bias.

DATABASES

PubMed

Scopus

Web of Science

Cochrane Central Register of Controlled Trials (CENTRAL)

NUMBER OF ARTICLES (FOR PRISMA CHART)

PubMed: 21

Scopus: 18

Web of Science: 22

Cochrane: 3

Total: 64

After duplicates removed: 54

Full-text articles assessed: 7

Included in the review: 4

QUALITY ASSESSMENT TOOL

Bias assessment was conducted using the Cochrane Risk of Bias Tool for randomized controlled trials (ROB2).
